# Supplementary material for: Genetic Diversity, Population Structure and Linkage Disequilibrium Analyses in Tropical Maize Using Genotyping by Sequencing
Source: Plants (Basel). 2022 Mar 17;11(6):799. doi: 10.3390/plants11060799 (PMC8955159; doi:10.3390/plants11060799)
Supplement: Supplementary file 1 [file plants-11-00799-s001.zip › Figure S1_Cluster and PCA Plots based on seven variables.pdf]

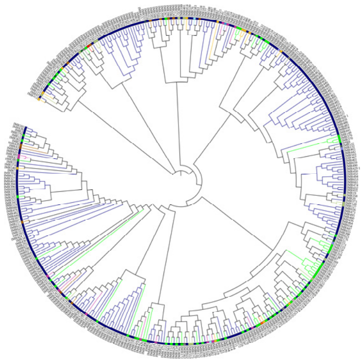

Variable 1. Clustering represented based on centers

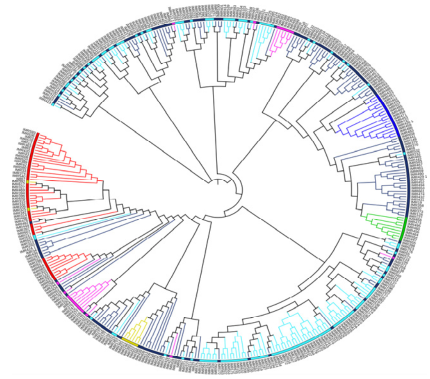

Variable 2. Clustering based on population structure

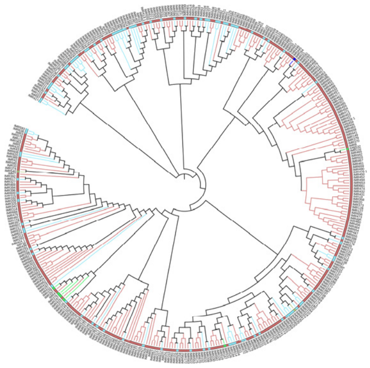

Variable 3. Clustering based on type of corn

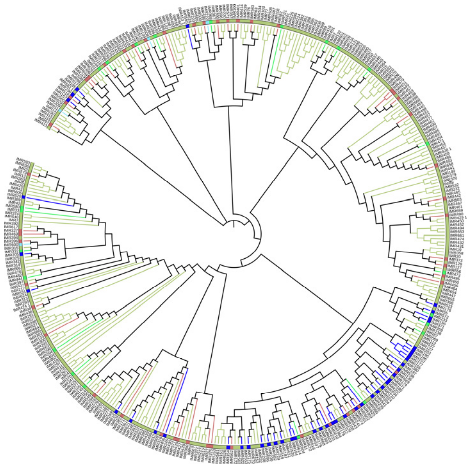

Variable 4. Clustering represented based on kernel colours

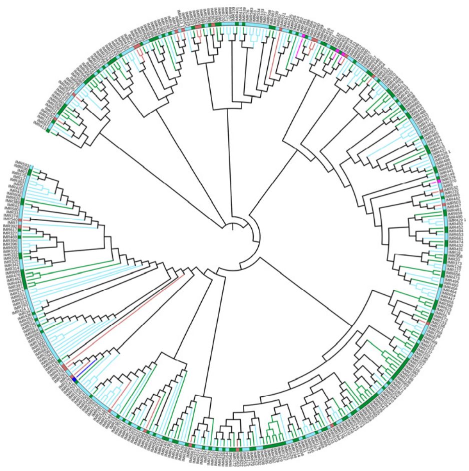

Variable 5. Clustering represented based on maturity

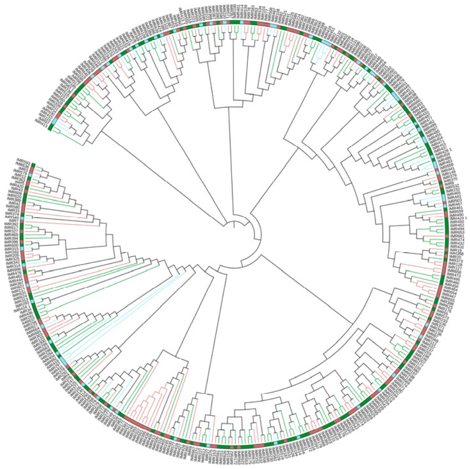

Variable 6. Clustering represented based on plant height

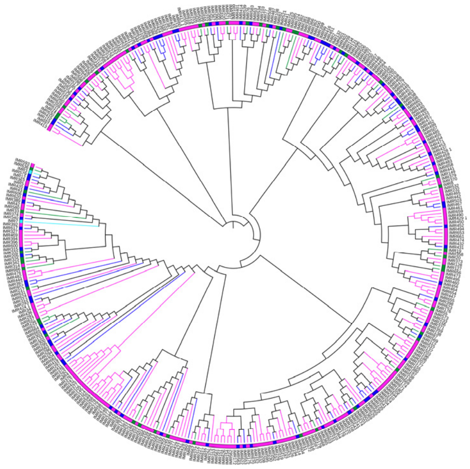

Variable 7. Clustering represented based on ear height

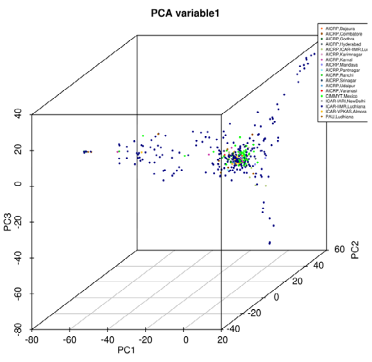

Variable 1. PCA represented based on the centers

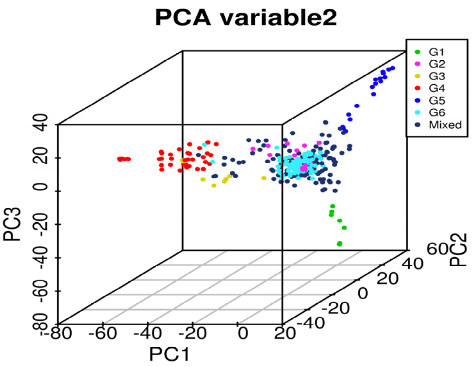

Variable 2. PCA represented based on population structure

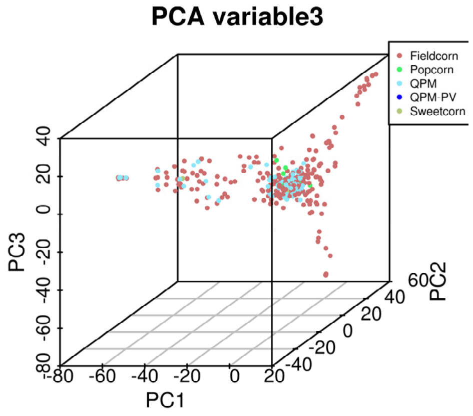

Variable 3. PCA represented based on type of corn

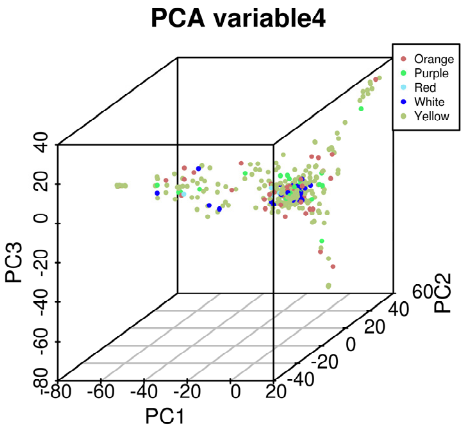

Variable 4. PCA represented based on kernel colour

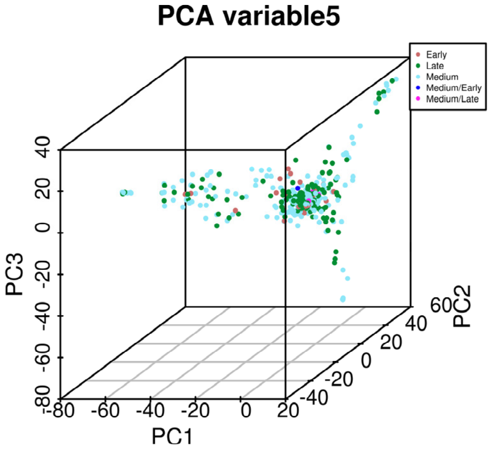

Variable 5. PCA represented based on maturity

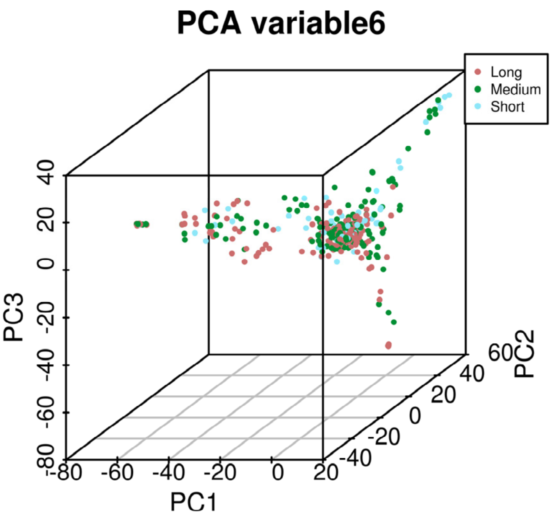

Variable 6. PCA represented based on based plant height

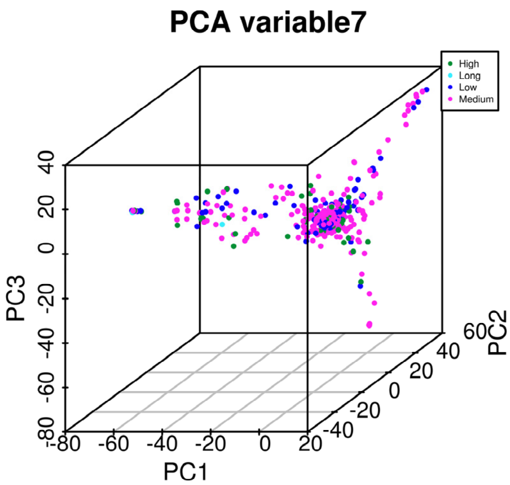

Variable 7. PCA represented based on based ear height placement

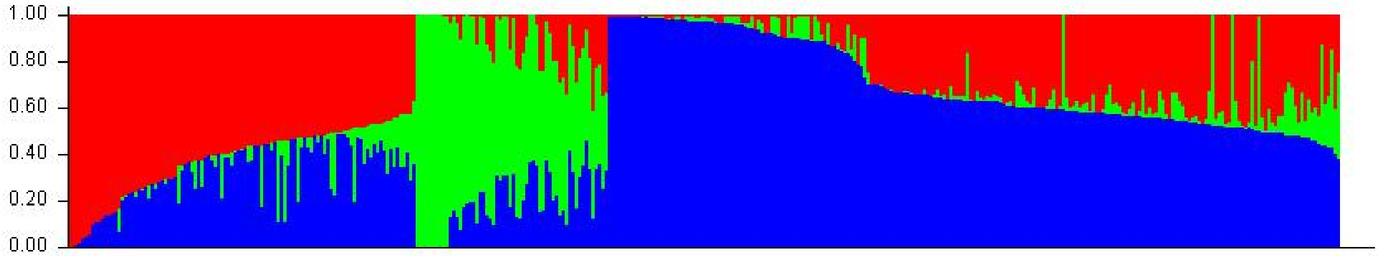

Population structure of 384 maize inbred lines revealed by 60277 SNP markers.

Figure S1. Different phylogenetic trees and PCA plots for 384 lines genotyped using 60,277 SNPs and represented based on seven variables such as center, population structure, types of corn, kernel colour, maturity group, plant height and ear height placement.
