# Supplementary material for: Genetic Diversity, Population Structure and Linkage Disequilibrium Analyses in Tropical Maize Using Genotyping by Sequencing
Source: Plants (Basel). 2022 Mar 17;11(6):799. doi: 10.3390/plants11060799 (PMC8955159; doi:10.3390/plants11060799)
Supplement: Supplementary file 1 [file plants-11-00799-s001.zip › Figure S2.pdf]

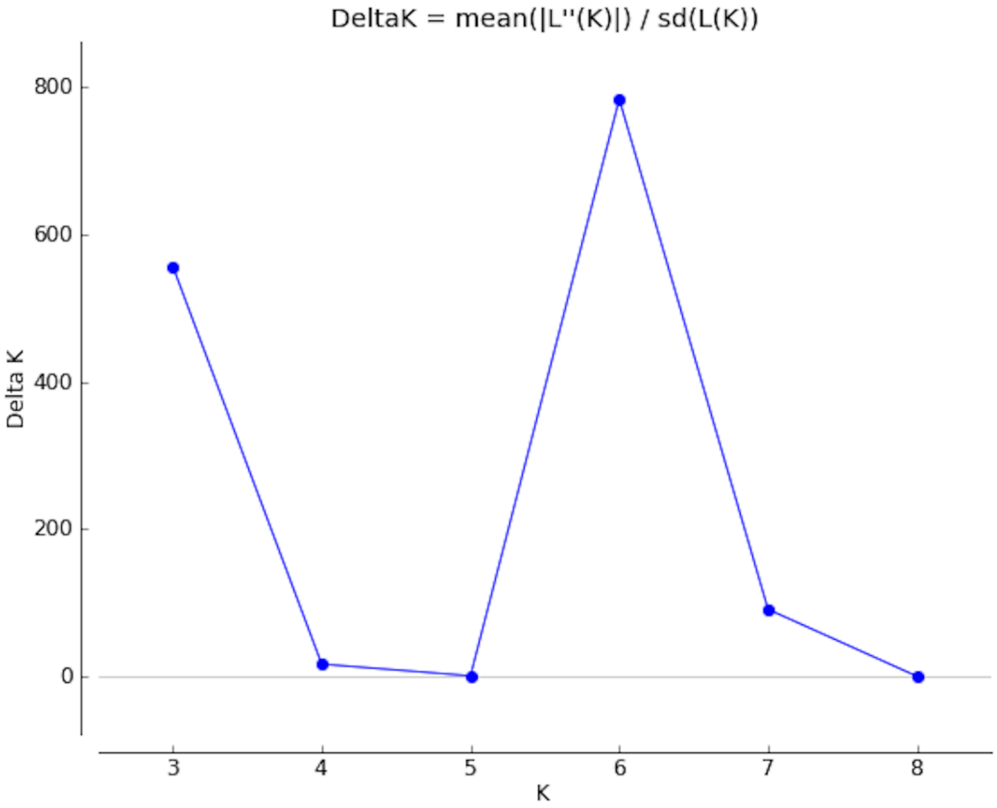

Figure S2. A lot of ad hoc statistics ΔK computed from changes of LnP(D) between K = 2 to K = 9. The highest peak at K = 6 suggests six sub-populations (groups) in the panel.
