# Supplementary material for: Genetic Diversity, Population Structure and Linkage Disequilibrium Analyses in Tropical Maize Using Genotyping by Sequencing
Source: Plants (Basel). 2022 Mar 17;11(6):799. doi: 10.3390/plants11060799 (PMC8955159; doi:10.3390/plants11060799)
Supplement: Supplementary file 1 [file plants-11-00799-s001.zip › Table S5.pdf]

Table S5. Mean LD (r^2) values across the ten maize chromosomes at different physical distances and MAF ≥ 0.05.

Distance (kb)	Chr.1	Chr.2	Chr.3	Chr.4	Chr.5	Chr.6	Chr.7	Chr.8	Chr.9	Chr.10	Over all mean LD
0-0.1	0.70	0.67	0.69	0.68	0.70	0.66	0.68	0.71	0.67	0.74	0.69
0.1-0.2	0.68	0.41	0.60	0.51	0.44	0.58	0.44	0.41	0.50	0.30	0.49
0.2-0.3	0.66	0.65	0.68	0.67	0.71	0.64	0.67	0.67	0.63	0.75	0.67
0.3-0.4	0.51	0.41	0.60	0.51	0.44	0.58	0.44	0.41	0.50	0.30	0.47
0.4-0.6	0.52	0.45	0.58	0.48	0.61	0.52	0.55	0.44	0.56	0.57	0.53
0.6-1.0	0.59	0.47	0.57	0.53	0.55	0.56	0.55	0.51	0.57	0.44	0.53
1.0-1.5	0.45	0.48	0.54	0.52	0.60	0.45	0.53	0.48	0.42	0.60	0.51
1.5-2.0	0.59	0.51	0.61	0.47	0.53	0.55	0.57	0.46	0.50	0.48	0.53
2.0-5.0	0.51	0.49	0.58	0.57	0.64	0.52	0.52	0.58	0.53	0.59	0.55
5.0-10.0	0.58	0.51	0.55	0.53	0.55	0.49	0.52	0.56	0.52	0.53	0.53
10.0-100.0	0.52	0.46	0.51	0.48	0.54	0.44	0.46	0.56	0.43	0.53	0.49
100.0-1000.0	0.42	0.34	0.47	0.36	0.48	0.32	0.40	0.50	0.32	0.44	0.40
1000.0-5000.0	0.31	0.26	0.45	0.24	0.44	0.21	0.32	0.40	0.23	0.36	0.32
5000.0-10000.0	0.22	0.21	0.41	0.20	0.28	0.19	0.30	0.25	0.21	0.28	0.25
10000.0-100000.0	0.17	0.19	0.26	0.18	0.17	0.17	0.21	0.16	0.20	0.20	0.19
>100000.0	0.14	0.15	0.16	0.15	0.15	0.16	0.15	0.14	0.13	0.16	0.15
Mean	0.47	0.42	0.52	0.44	0.49	0.44	0.46	0.45	0.43	0.45	0.46
